# Advancing Scaffold Architecture for Bone Tissue Engineering: A Comparative Study of 3D-Printed β-TCP Constructs in Dynamic Culture with pBMSC

**DOI:** 10.3390/jfb16090327

**Published:** 2025-09-04

**Authors:** Yannick M. Sillmann, Ana M. P. Baggio, Pascal Eber, Benjamin R. Freedman, Cynthia Liu, Youssef Jounaidi, Alexander Schramm, Frank Wilde, Fernando P. S. Guastaldi

**Affiliations:** 1 Division of Oral and Maxillofacial Surgery, Department of Surgery, Massachusetts General Hospital, Boston, MA 02114, USA; ana.baggio@unesp.br (A.M.P.B.); eberpascal.94@gmail.com (P.E.); 2 Harvard School of Dental Medicine, Boston, MA 02115, USA; 3 Department of Oral and Plastic Maxillofacial Surgery, University Hospital Ulm, 89081 Ulm, Germany; alexander.schramm@uni-ulm.de (A.S.); frank.wilde@uni-ulm.de (F.W.); 4 John A. Paulson School of Engineering and Applied Sciences, Harvard University, Cambridge, MA 02134, USA; bfreedm2@bidmc.harvard.edu (B.R.F.); cliu0715@gmail.com (C.L.); 5 Wyss Institute for Biologically Inspired Engineering at Harvard University, Boston, MA 02215, USA; 6 Department of Anesthesia, Critical Care and Pain Medicine, Massachusetts General Hospital, Harvard Medical School, Boston, MA 02114, USA; yjounaidi@mgh.harvard.edu; 7 Department of Oral and Plastic Maxillofacial Surgery, Military Hospital Ulm (Academic Hospital of the University of Ulm), 89081 Ulm, Germany

**Keywords:** scaffold pore size, bone tissue engineering, β-tricalcium phosphate, dynamic culture, osteogenic differentiation, bioreactor, 3D printing, mesenchymal stem cells, regenerative medicine, oral and maxillofacial surgery

## Abstract

Scaffold architecture is a key determinant of cell behavior and tissue regeneration in bone tissue engineering, yet the influence of pore size under dynamic culture conditions remains incompletely understood. This study aimed to evaluate the effects of scaffold pore size on osteogenic differentiation of porcine bone marrow-derived mesenchymal stem cells (pBMSCs) cultured in a rotational oxygen-permeable bioreactor system (ROBS). Three-dimensionally (3D) printed beta-tricalcium phosphate (β-TCP) scaffolds with pore sizes of 500 µm and 1000 µm were seeded with pBMSC and cultured for 7 and 14 days under dynamic perfusion conditions. Gene expression analysis revealed significantly higher levels of osteogenic markers (Runx2, BMP-2, ALP, Osx, Col1A1) in the 1000 µm group, particularly at the early time point, with the later-stage marker Osteocalcin (Ocl) rising faster and higher in the 1000 µm group, after a lower expression at 7 days. ALP activity assays corroborated these findings. Despite having lower mechanical strength, the 1000 µm scaffolds supported a homogeneous cell distribution and high viability across all regions. These results suggest that larger pore sizes enhance early osteogenic commitment by improving nutrient transport and fluid flow in dynamic culture. These findings also support the use of larger-pore scaffolds in bioreactor-based preconditioning strategies and underscore the clinical importance of promoting early osteogenic differentiation to reduce in vitro culture time, an essential consideration for the timely preparation of implantable grafts in bone tissue engineering.

## 1. Introduction

The reconstruction of critical-sized bone defects in the craniofacial region remains one of the most demanding challenges in oral and maxillofacial surgery [1,2]. These defects, often resulting from high-energy trauma, congenital anomalies, or oncologic and non-oncologic resections, exceed the intrinsic regenerative capacity of bone and necessitate surgical reconstruction [2,3,4]. In particular, mandibular defects are associated with significant functional and aesthetic impairments, including compromised mastication, speech, and facial symmetry [1,2,3,4].

Autologous bone grafting, typically involving vascularized free flaps such as the fibula, iliac crest, or scapula, is currently considered the gold standard for segmental mandibular reconstruction [1,2,4,5]. While effective in restoring form and function, these procedures are constrained by several well-recognized limitations. These include limited graft availability, prolonged operative/anesthesia time, and significant donor site morbidity [2,6,7]. Moreover, the need for multiple surgical sites increases perioperative and postoperative complexity and patient burden [2,6].

Given these constraints, developing alternative strategies for bone regeneration is a priority in the field [5,8]. Bone tissue engineering (BTE) has emerged as a promising approach, offering the potential to regenerate osseous tissue through the combination of biocompatible scaffolds, osteogenic cells, and growth factors [5,9,10,11,12,13]. In this context, scaffold-based constructs are being increasingly investigated as customizable solutions for reconstructing load-bearing defects in the craniofacial skeleton [5,9,10,12].

These constructs are designed to provide a three-dimensional (3D) framework that supports cellular attachment, migration, and osteogenic differentiation while gradually degrading as new bone forms [9,11].

Advances in biomaterials and fabrication techniques, particularly additive manufacturing, have enabled the production of scaffolds with precisely controlled geometries and pore architectures [14,15,16,17]. Such customization is especially valuable in the reconstruction of irregular, load-bearing craniofacial defects where anatomical fidelity and mechanical integrity are essential [14,15,16,17]. Furthermore, combining these scaffolds with autologous mesenchymal stem cells and osteoinductive cues has shown promising results in preclinical models, supporting their potential as clinically translatable alternatives to autografts [5,8,9,10,18].

The regenerative performance of a scaffold is strongly influenced by its microarchitecture, particularly pore size, porosity, and interconnectivity [5,9]. These parameters affect not only the mechanical stability of the construct but also critical biological processes such as cell infiltration, vascularization, and nutrient diffusion [9,19,20]. Among them, pore size has emerged as a pivotal factor in directing bone formation [9,19].

Experimental studies have demonstrated that pore sizes within a specific range can enhance osteoblast attachment, matrix deposition, and neovascularization, whereas suboptimal dimensions may hinder cell migration or compromise scaffold integrity [9,19,21,22]. However, most of these investigations have been conducted under static culture conditions, where diffusion limitations and uneven nutrient gradients often obscure the role of scaffold architecture [23,24,25].

Dynamic culture systems, such as perfusion bioreactors, offer significant advantages compared to conventional static culture [23,24,25,26]. By providing continuous medium flow, they enhance nutrient transport, oxygenation, and metabolic waste removal throughout the entire scaffold, thereby preventing central necrosis and supporting homogeneous cell colonization [24,26,27]. Moreover, dynamic flow introduces mechanical cues, including shear stress, that are known to stimulate osteogenic signaling and accelerate lineage commitment [23,24,26]. Beyond improving cell survival, dynamic systems facilitate the generation of more physiologically relevant 3D constructs and have been proposed as an essential step in the preconditioning of implantable grafts, even enabling organoid-like tissue formation [23,25,26].

In this context, scaffold pore size plays a particularly important role under perfusion conditions. Larger pores can reduce flow resistance and allow deeper penetration of the culture medium, while smaller pores may enhance initial cell attachment but risk creating diffusion bottlenecks [23,26]. Thus, the interplay between pore size and fluid dynamics is likely to be a decisive factor in osteogenic differentiation outcomes when scaffolds are cultured dynamically.

The rise of modern additive manufacturing techniques has enabled the fabrication of scaffolds with highly controlled and reproducible architectures [15,16]. Among these, 3D-printed β-TCP has garnered particular attention due to its excellent biocompatibility, osteoconductivity, and resorbability [9,13,28,29]. Unlike traditional fabrication methods, 3D printing enables the precise modulation of pore geometry and spatial organization, allowing for a systematic investigation of microstructural parameters, such as pore size, under standardized conditions [9,15,16,29]. In the context of craniofacial reconstruction, β-TCP scaffolds offer the dual advantage of structural stability and gradual in vivo degradation, making them attractive candidates for large, load-bearing defects [9,28,29,30].

When combined with dynamic perfusion culture, these scaffolds provide a powerful experimental platform to dissect how specific architectural parameters, such as pore size, influence early osteogenic differentiation.

We deliberately selected comparatively large pore sizes, with the 1000 µm group representing an extreme case, to specifically test how dynamic perfusion interacts with macroporosity. While such dimensions may exceed the traditionally reported optimal ranges for craniofacial bone regeneration under static conditions [9], they allow us to evaluate whether enhanced flow and nutrient transport in a dynamic culture can offset these limitations and promote accelerated osteogenic differentiation.

This study aimed to investigate how scaffold pore size influences the early osteogenic differentiation of porcine bone marrow-derived mesenchymal stem cells (pBMSCs) in a dynamic culture environment. Two β-TCP scaffold groups, identical in material composition and overall design but differing in macroscopic pore size (500 µm vs. 1000 µm), were fabricated using additive manufacturing and subjected to mechanical and structural characterization to ensure comparability.

Osteogenic differentiation was assessed primarily through the analysis of key osteogenic marker genes and the activity of the enzyme alkaline phosphatase (ALP). We hypothesized that scaffolds with larger pores would promote greater and faster osteogenic gene expression under dynamic culture conditions by enhancing nutrient diffusion, oxygen availability, and metabolic waste removal.

## 2. Materials and Methods

### 2.1. Fabrication of the Scaffolds

The scaffolds [n = 82; 500 µm (n = 41) and 1000 µm (n = 41)] were designed by the Skeletal Biology Research Center (SRBC) at Massachusetts General Hospital (Boston, MA, USA) and manufactured by KLS Martin (Tuttlingen, Germany). The 3D-printing procedure LithaBone TCP 300 by Lithoz (Vienna, Austria) was implemented to fabricate scaffolds, consisting of ≥95% purity beta-tricalcium-phosphate (β-TCP), measuring 10 mm × 10 mm × 8 mm while containing two different microarchitectures (500 µm and 1000 µm) of interconnected pores and a strut diameter of 0.5 mm.

Post-printing thermal processing (debinding and sintering) was performed by the manufacturer (KLS Martin; Tuttlingen, Germany) according to the established Lithography-based Ceramic Manufacturing (LCM) protocol for LithaBone TCP 300. LCM processing removes the organic binder and densifies the β-TCP ceramic. By varying sintering parameters (1000–1200 °C), relative densities up to 98% are achievable with this material. Both pore-size groups in this study were processed identically. The scaffolds were generously donated to the SRBC by KLS Martin. The scaffold architecture, including pore geometry and strut diameter, was adapted from the validated design framework described by Roque et al. [31]. The total sample size (n = 82) was determined based on commonly reported group sizes in comparable in vitro scaffold studies [5,8,32], as well as logistical constraints associated with scaffold fabrication, perfusion bioreactor operation, and multi-parametric downstream analysis. The study was designed as an exploratory investigation to identify biologically meaningful differences rather than to test a specific statistical hypothesis.

### 2.2. Physicochemical, Morphological, and Mechanical Characterization

The morphology of the 3D printed β-TCP scaffolds was analyzed using a Field Emission Scanning Electron Microscope (FESEM). Energy Dispersive X-ray Spectroscopy (EDX) was used to analyze the elemental composition of the scaffolds. The microarchitecture of the different scaffolds was analyzed by microcomputed tomography (micro-CT). The compressive mechanical properties (mechanical strength) of the scaffolds were determined using a mechanical testing machine (n = 5 per group; total = 10 scaffolds).

#### 2.2.1. Field Emission Scanning Electron Microscope (FESEM)

To analyze the morphological and topographical characteristics of the scaffolds, they were scanned via a Field Emission Scanning Electron Microscope (FESEM) (Phenom ProX table-top SEM, ThermoFisher, Waltham, MA, USA). A sample scaffold from each group was dried in a vacuum chamber for 15 min prior to scanning. Each scaffold was loaded into the vacuum chamber of the FESEM, and the acceleration voltage was set to 5 kV, with a probe current of 60 pA.

Imaging was performed at different magnifications. The lowest magnification possible was 112× for 500 µm scaffolds and 80× for the 1000 µm scaffolds. Both groups were imaged at 500×, 1000×, 5000×, and 10,000×.

#### 2.2.2. Energy Dispersive X-Ray Spectroscopy (EDS)

To analyze the elemental composition of the scaffolds, energy-dispersive X-ray spectroscopy (EDS) was implemented. Three fields of interest were randomly selected on each scaffold surface and analyzed using an acceleration voltage of 15 kV, a backscattered electron detector (BSD), and a field of view of 53.7 µm. Spectra with characteristic elemental peaks, indicating the chemical composition of the scaffold material, were collected.

#### 2.2.3. Micro-Computed Tomography

The porosity, mean pore size, and mineral density of the scaffolds were measured via µ-CT imaging (Scanco mCT40, Scanco Medical AG, Bruettisellen, Switzerland). The scaffolds (n = 3 per group) were immersed in phosphate-buffered saline (PBS) and scanned using a high-resolution cone-beam CT scanner with a voxel size of 36 µm, an X-ray tube potential of 70 kVp (peak), an intensity of 114 mAs, and an integration time of 300 ms. The generated images represent horizontal slices of the scaffolds.

The scaffold porosity (Φ) was evaluated by dividing the void volume by the total scaffold volume (Φ = V_void_/V_total_). Through measuring the outer dimensions of the scaffold, the total volume (V_total_) was measured (V_total_ = V_void_ + V_material_). The scaffold void volume (V_void_) was calculated by subtracting the measured scaffold material volume (V_material_) from the total volume (V_void_ = V_total_ − V_material_). The overall mineral density was analyzed via the Scanco μCT Evaluation Software (version 6.5).

#### 2.2.4. Mechanical Test

To evaluate the mechanical stability of the scaffolds under load, uniaxial compression testing was performed using a static testing machine (Instron 5944, Instron, Norwood, MA, USA). The scaffolds were placed between two steel plates and compressed at a constant crosshead speed of 10 mm/min until material failure. During the test, the machine automatically recorded the maximum compressive force. The scaffolds were tested until material failure occurred. The cross-sectional area of the scaffolds and the maximum compressive force through the machine were measured to analyze the maximum stress, toe stiffness, linear stiffness, toe modulus, linear modulus, and transition strain of the groups. A total of n = 5 scaffolds per group were analyzed.

### 2.3. In Vitro Study

#### 2.3.1. Cell Sources and Culture

The pBMSCs cell line was obtained from previously isolated and cryopreserved cells generated in the study by Mueller et al. [5], in which pBMSCs were harvested from a 6-month-old female Yucatan minipig (Sinclair Bio Resources LLC., Windham, ME, USA) under a Massachusetts General Hospital (MGH) Institutional Animal Care and Use Committee (IACUC)-approved protocol (2017N000073) following the procedure described by Abukawa et al. [8]. The pBMSCs were seeded in 75 cm^2^ tissue culture flasks with a vented cap (Thermo Fisher Scientific, Waltham, MA, USA) and cultured in 15 mL of basal media consisting of Dulbecco’s modified eagle media (DMEM) (Invitrogen, Waltham, MA, USA) supplemented with 10% of fetal bovine serum (FBS, Gibco, Thermo Fisher Scientific, Waltham, MA, USA), 1% of modified eagle media non-essential aminoacids solution (MEM NEAA, Gibco, Thermo Fisher Scientific, Waltham, MA, USA), 1% of antibiotic/antimycotic solution (penicillin 10,000 U/mL, streptomycin 10,000 µg/mL, amphotericin B 25 µg/mL; Sigma-Aldrich, Burlington, MA, USA) under standard conditions (5% CO_2_, 95% humidity, and 37 °C). The medium was changed every second day. The cells were cultured until passage 4 to 7, and once 90% confluence was observed under a light microscope, the cells were sub-cultured using 0.25% Trypsin-EDTA (Invitrogen, Waltham, MA, USA) and resuspended in culture media. Finally, 1.5 × 10^6^ cells were used to seed each β-TCP scaffold.

#### 2.3.2. Osteogenic Differentiation Assay of pBMSC

To evaluate the osteogenic differentiation potential of the stem cells, an osteogenic differentiation assay was conducted. A culture of pBMSCs was seeded at a density of 10,000 cells per 25 cm^2^ flask. The cells were incubated under the conditions described above, with an osteogenic differentiation medium for 14 days (media change every 3 days). For control purposes, duplicate cultures were maintained in regular medium without osteogenic supplements. The osteogenic differentiation medium consisted of the standard culture medium supplemented with 10 mM/L β-glycerophosphate, 100 nM/L dexamethasone, and 50 μg/mL ascorbic acid. After 14 days, the cell cultures were gently washed with PBS and fixed with absolute ethanol. To assess cell mineralization capacity, the cultures were stained with 2% Alizarin Red (Lifeline^®^ Cell Technology, San Diego, CA, USA), following the manufacturer’s protocol. This assay was performed as a validation step to confirm that the pBMSCs used in this study retained their ability to undergo osteogenic differentiation and deposit mineralized matrix effectively.

#### 2.3.3. Cell Seeding, Construct Preparation, and Osteogenic Differentiation

The scaffolds were sterilized under UV light for 24 h prior to cell-seeding. The scaffolds were placed in a 9-well plate (Thermo Fisher Scientific, USA), and the resuspended cells were equally distributed on the scaffold surface. A total of 1.5 × 10^6^ pBMSC were applied dropwise to the upper scaffold surface and macropores to facilitate cell seeding throughout the scaffold. The seeded scaffolds were incubated for 4 h at 37 °C to allow cells to adhere to the scaffold surface.

After the incubation period, osteogenic differentiation of the constructs (construct = scaffold seeded with pBMSC) commenced. Each construct was placed in a vented Falcon bioreactor tube (Thermo Fisher Scientific, USA) containing 20 mL of osteogenic medium. For further incubation, vented tubes were placed in a custom-built Rotational Oxygen-Permeable Bioreactor System (ROBS) [5,8,33]. The ROBS was developed for previous studies by the Skeletal Biology Research Center [5,8,33,34]. The ROBS consisted of a spinning cylinder, which held the vented tubes inside an incubator at a constant speed of 4 rpm, at 37 °C, 5% CO_2_, and 42% humidity. Constructs of both scaffold groups were kept inside the ROBS for 7 or 14 days. The osteogenic media were changed on Days 3, 7, and 10.

#### 2.3.4. Cell Proliferation and Viability on the Scaffold Surface

##### DAPI Staining

Cell density within the constructs was assessed via 4′,6-diamidino-2-phenylindole (DAPI) staining after 7 and 14 days of osteogenic differentiation. Constructs were fixed in 10% formalin (Sigma-Aldrich Burlington, MA, USA) for 20 min and washed twice with PBS. Subsequently, the scaffolds were incubated with a 300 nM DAPI solution for 5 min at room temperature, followed by additional PBS washes to remove any unbound stain. Each construct was bisected in the middle, and the internal cross-sectional plane was imaged using a confocal laser scanning microscope (Nikon Eclipse Ti2, Nikon, Tokio, Japan) equipped with a 405 nm excitation laser and a DAPI emission filter.

**Figure 1:**
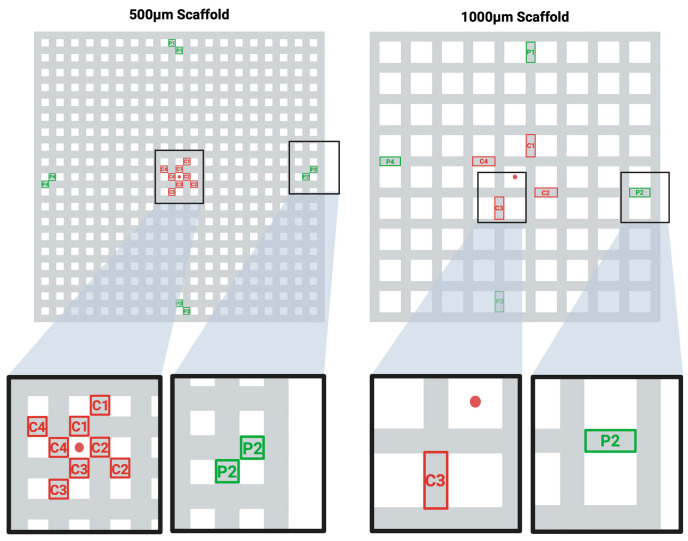
Selected Areas of Interest for the DAPI-stained Cell Density Analysis. Schematic illustration of the middle plane in the two scaffold designs (500 µm group/1000 µm group). The standardized regions of interest for each design are depicted in an orientation where the four central regions (red) (C1–C4) and the four peripheral regions (green) (P1–P4) are analyzed. The DAPI-stained nuclei were counted in each region of interest in z-stacks of n = 120 images. The center of the scaffold planes is marked with a red dot.

Quantitative analysis was performed by counting cell nuclei within eight standardized regions (total area: 500 µm × 1000 µm per construct), four located at the construct center and four bordering the peripheral surface (see Figure 1). To ensure area consistency across groups despite differing strut dimensions, the analysis included either two adjacent struts of 500 µm × 500 µm each (500 µm group) or a single strut of 500 µm × 1000 µm (1000 µm group). Each region was imaged as a Z-stack (n = 120 images per stack).

Image analysis and quantification were conducted using ImageJ 2 Version 1.54p (NIH, Bethesda, MD, USA). For each of the eight defined regions of interest (ROIs), the corresponding Z-stack (120 slices per ROI) was loaded and processed individually. To enable accurate cell counting despite overlapping nuclei in projection images, each Z-stack was analyzed slice by slice. After converting the image stacks to 8 bits and applying contrast normalization, a consistent binary threshold was applied across all slices. For each slice, a manually defined ROI, either central or peripheral, was applied using the ROI Manager, and the number of DAPI-positive nuclei was quantified using the “Analyze Particles” function, excluding noise by setting a size filter (50–Infinity pixels^2^). For each ROI, counts from all 120 slices were summed to yield the total number of nuclei. Central and peripheral values were averaged separately for each construct to facilitate spatial comparison of cell distribution. For each group and time point, we analyzed n = 3 constructs.

##### Live/Dead Staining

Cell survival and viability were analyzed according to the manufacturer’s instructions using a LIVE/DEAD Viability/Cytotoxicity Kit (ThermoFisher, Waltham, MA, USA). After 7 and 14 days in the ROBS, the viability of the cells covering the two different constructs was assessed. The constructs were washed twice with PBS to remove any remaining media. Afterwards, the constructs were covered with a 2 µM Calcein AM (green fluorescence) and 4 µM Ethidium homodimer-1 (EthD-1) (red fluorescence) solution (diluted in 10 mL of PBS) and incubated, protected from light, at 37 °C for 40 min (5% CO_2_, 42% humidity). The constructs were immediately evaluated using a confocal microscope (Nikon Eclipse Ti2, Nikon, Japan) with the green (488.0 nm) and red (561.0 nm) channels. The constructs were cut in half, and the inner surface of each construct was evaluated. For each group, the experiment was carried out in triplicate.

##### Alkaline Phosphatase (ALP) Activity

The Ab83369 Alkaline Phosphatase Assay Kit (Colorimetric) (Abcam, Cambridge, MA, USA) was used to analyze the ALP activity of the constructs according to the manufacturer’s instructions, using p-nitrophenyl phosphate (pNPP) as the substrate and ALP as the standard. The plate was incubated at 25 °C for 60 min while being protected from light. A stop solution was added to each well before the optical density (OD) was measured.

In each group, n = 5 constructs were osteogenically differentiated in the ROBS, and the media were collected after 7 and 14 days of differentiation. Through the media-changing intervals (described above), at both time points, the cell-media remained in the tube containing the construct for 4 days before it was collected.

The OD was measured at 405 nm using a microplate reader (SpectraMax M5, Molecular Devices, San Jose, CA, USA). A standard curve was calculated, and the ALP activity per media sample was measured four times per sample and averaged. For ALP enzymatic activity and DNA quantification, five biological replicates per group were used to ensure sufficient statistical power while balancing the feasibility of scaffold culture and biochemical analysis under dynamic conditions [32,35].

##### Real-Time Polymerase Chain Reaction (RT-PCR)

To extract the total RNA from the cells covering the constructs, after 7 and 14 days in the ROBS, the constructs were soaked in 1 mL of TRIzol Reagent (guanidinium thiocyanate) (Invitrogen, Waltham, MA, USA) and ground to a paste in a sterile ceramic grinder. The liquid supernatant was pipetted into an Eppendorf tube, mixed with 0.2 mL of Chloroform (Thermo Fisher Scientific, USA), and centrifuged at 12,000× *g* for 15 min. The top aqueous phase was transferred into another Eppendorf tube, mixed with 0.5 mL of isopropyl alcohol, incubated for 10 min at room temperature, and centrifuged (12,000× *g*/min) again for 10 min. The resulting RNA pellet was washed with 1 mL of 75% ethanol and centrifuged again (7000× *g*/min; 5 min). The RNA pellet was briefly dried before being dissolved in 50 µL of RNAse-free, deionized, diethylpyrocarbonate (DEPC)-treated water. The amount of extracted RNA was determined using a UV–VIS Spectrophotometer (UV-1280, Shimadzu, Kyoto, Japan), and the samples were stored at −80 °C.

In a second step, 1 µg of RNA per sample was converted into cDNA using an enzyme (Reverse Transcriptase) to perform unbiased cDNA synthesis. The protocol was implemented as advised by the manufacturer (SMARTScribe, TaKaRa Bio Inc., Shiga, Japan). A total of 1 µg of RNA per sample and 1 µL of 20µM primer stock (OligoDT, Invitrogen, Waltham, MA, USA) was added to an RNA-free 0.2 mL Bio-Rad PCR Tube (Bio-Rad Laboratories, Hercules, CA, USA), where the final volume was topped up to 10 µL with RNase-free DEPC water. The mixture was heated to 72 °C for 3 min and then placed on ice afterwards. Then, 4 µL of 5X First-Strand Buffer, 2 µL dNTP Mix, 2 µL 20 mM DTT, and 2 µL (100 U/µL) SMART ScribeRT were added to the PCR tubes. The samples were then incubated at 42 °C for 90 min. The reaction was terminated by heating the samples to 70 °C for 15 min. Afterwards, the samples were stored at −20 °C.

In the third step, qRT-PCR was performed using the cDNA with a Power Track^TM^ SYBR Green Master Mix (Applied Biosystems^TM^, Thermo Fisher Scientific, Waltham, MA, USA) in a 7500 Fast Real-Time PCR System (Applied Biosystems^TM^, Thermo Fisher Scientific, Waltham, MA, USA). The cDNA samples were diluted 1:5 with DPEC water, and 6 µL per sample were pipetted into each designated well of a 96-well qPCR plate (Thermo Fisher Scientific, USA). Additionally, 10 µL of SYBR Green Master Mix and 2 µL of DPEC water were added to each well. Then, 1 µL of forward and 1 µL of reverse primer were added to the appropriate wells of the plate. The PCR primers to assess osteogenic differentiation were designed using MacVector version 18.7 software (MacVector, Apex, NC, USA) and synthesized by IDT DNA Technologies (Coralville, IA, USA). They are detailed in Table 1. The plates were briefly centrifuged and analyzed via the 7500 Fast Real-Time PCR System.

For each sample (n = 5 per group), the experiments were performed in triplicate and included a negative control group. The relative standard curve method (2^−ΔΔCT^) was used to determine the relative mRNA expression, with the endogenous Glyceraldehyde 3-phosphate dehydrogenase (GAPDH) housekeeping gene serving as a positive control. To provide a negative control group for osteogenic differentiation, n = 5 cell cultures of the same pBMSCs, cultured in regular medium, were analyzed. For quantitative PCR, a group size of n = 3–5 per condition is consistent with standard practice in gene expression studies involving stem cell differentiation on 3D scaffolds, where technical variability is low and fold changes in expression are typically robust [32,35].

### 2.4. Statistical Analysis

The statistical analysis for all results was performed with Microsoft Excel version 16.84 (Microsoft, Redmond, WA, USA) and GraphPad Prism version 10.2.3 (San Diego, CA, USA). The data were descriptively analyzed and expressed as the mean and standard deviation (±SD) for all experiments. Statistical significance was determined using either an unpaired *t*-test or Welch’s test. A probability value (*p*) of ˂0.05 was considered statistically significant. To express a level of significance, the terms * = *p* ˂ 0.05; ** = *p* ˂ 0.01; *** = *p* ˂ 0.001; **** = *p* ˂ 0.0001 were used.

## 3. Results

### 3.1. Physicochemical, Morphological, and Mechanical Characterization

#### 3.1.1. Scaffolds

**Figure 2:**
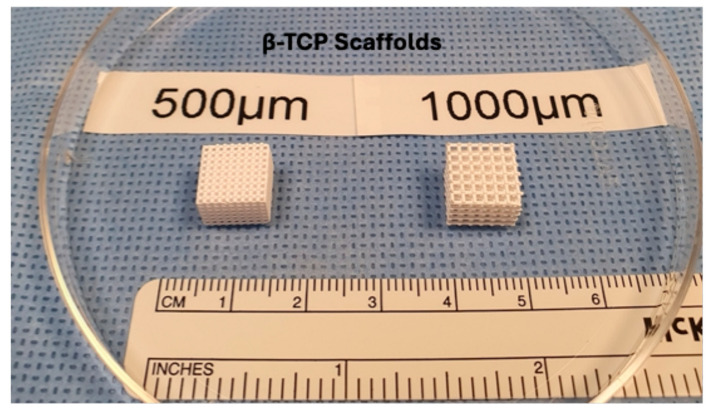
Left: Scaffold from the 500 µm group; right: Scaffold from the 1000 µm group.

The β-TCP scaffolds were 3D-printed into cubes measuring 10 mm × 10 mm × 8 mm, with a strut size of 0.5 mm and two different architectures (macropore sizes: 500 µm or 1000 µm). The scaffolds are shown in Figure 2.

#### 3.1.2. Field Emission Scanning Electron Microscopy (FESEM)

FESEM analysis confirmed the high morphological precision and reproducibility of the 3D-printed β-TCP scaffolds across both pore size groups. At low magnifications (112× for the 500 µm group and 93× for the 1000 µm group), the macroporous architecture appeared well defined and consistent with the design parameters. The pore geometries were regularly distributed, and the pore diameters closely matched their intended dimensions, underscoring the high accuracy of the additive manufacturing process.

At intermediate magnifications (500× to 1000×), both groups displayed a uniform strut morphology with well-preserved interconnections between pores. The strut surfaces were consistently rough and granular, a surface characteristic known to promote cell adhesion. Notably, the morphology and surface texture of the struts were similar between the 500 µm and 1000 µm groups, reflecting identical material composition and identical post-printing thermal processing.

At higher magnifications (5000× and 10,000×), fine microporosities and sintering-induced grain structures were observed on the strut surfaces. These nano-scale surface irregularities were homogeneously distributed in both scaffold types, suggesting favorable conditions for protein adsorption and initial cell anchorage. Importantly, no defects such as cracks, delamination, or uneven surface regions were detected, indicating excellent structural integrity and surface continuity.

**Figure 3:**
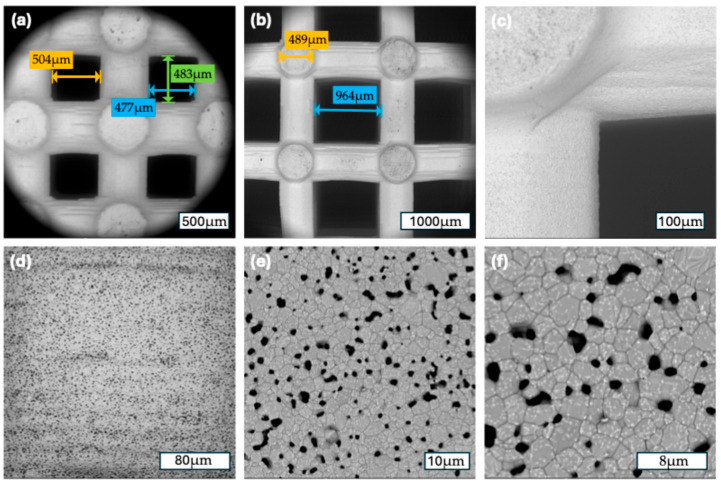
(**a**) A 500 µm scaffold group: 112×, measurements of the macropore diameter; (**b**); a 1000 µm scaffold group: 80×, measurements of the macropore and strut diameter (**c**) a 1000 µm scaffold group: 500×, corner of a macropore; (**d**) a 500 µm scaffold group: 1000×, surface of a strut; (**e**) a 1000 µm scaffold group: 5000×, surface of a strut (**f**) a 1000 µm scaffold group: 10,000×, surface of a strut.

Overall, FESEM imaging validated the high-resolution fidelity of the 3D-printing process and confirmed that both scaffold types possessed identical surface features and compositional characteristics, differing solely in macropore size (Figure 3).

#### 3.1.3. Energy Dispersive X-Ray Spectroscopy (EDS)

The EDS spectra confirmed the presence of β-TCP (Ca, P, and O). The mean atomic concentration for oxygen (O) was 80.36 cm^−3^ (SD: 0.21 cm^−3^), for calcium (Ca) 11.27 cm^−3^ (SD: 0.26 cm^−3^), and for phosphorus (P) 8.37 cm^−3^ (SD: 0.31 cm^−3^). The atomic weight concentration was 64.40 kg^−1^ (SD: 0.29 kg^−1^) for O, 22.62 kg^−1^ (SD: 0.48 kg^−1^) for Ca, and 12.98 kg^−1^ (SD: 0.48 kg^−1^) for P. The elemental mapping showed a homogeneous distribution of the elements throughout the scaffolds.

#### 3.1.4. Micro-Computed Tomography

**Figure 4:**
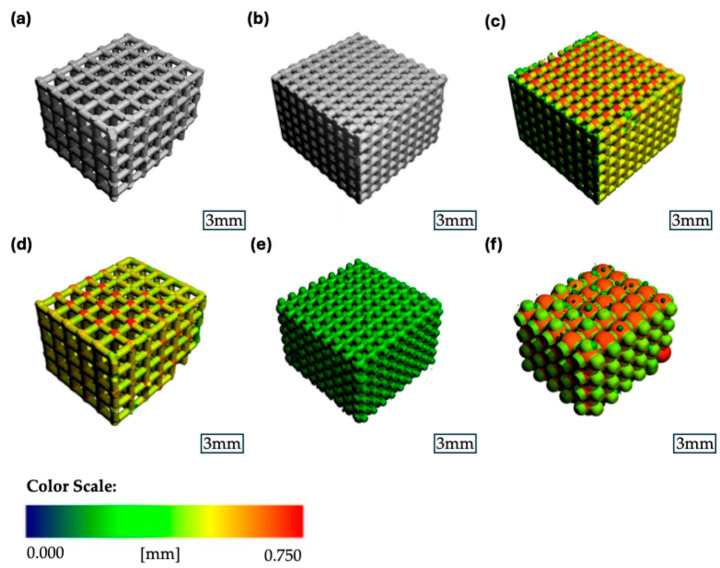
Representative µCT images of the β-TCP scaffolds: (**a**) binary segmented scaffold 1000 µm group; (**b**) binary segmented scaffold 500 µm group; (**c**) strut thickness heatmap scaffold 500 µm group; (**d**) strut thickness heatmap scaffold 1000 µm group; (**e**) pore size heatmap scaffold 500 µm group; (**f**) pore size heatmap scaffold 1000 µm group.

Both scaffold groups were analyzed for their porosity, mean pore size, strut thickness, and mineral density, and the results are shown in Figure 4.

**Figure 5:**
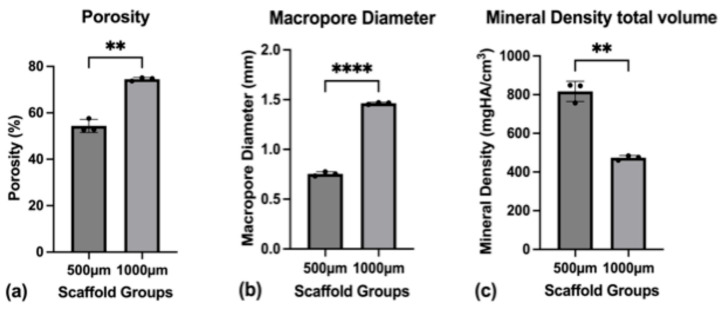
(**a**) Porosity of the scaffolds as a whole; (**b**) Macropore diameter (3D volume) measured through micro-CT; (**c**) Mineral density for the total scaffold volume. To express a level of significance, the terms; ** = *p* ˂ 0.01 and **** = *p* ˂ 0.0001 were used.

The porosity of the 500 µm group had a mean value of 54.4% (SD ± 2.8%), while the 1000 µm group showed a significantly larger porosity of 74.5% (SD ± 0.71%) (*p* = 0.0041) (Figure 5a). The mean pore diameter for the 500 µm group was 0.754 mm (SD ± 0.021 mm), while for the 1000 µm group, it was significantly larger at 1.464 mm (SD ± 0.010 mm) (*p* < 0.0001) (Figure 5b). The average thickness of the scaffold’s struts was measured for both groups, showing a mean strut-diameter of 0.556 mm (SD ± 0.012 mm) for the 500 µm group and 0.538 mm (SD ± 0.007 mm) for the 1000 µm group (*p* = 0.1045). The connectivity density, describing the number of strut intersections per 1 mm^3^, was 2.07/mm^3^ (SD ± 0.11/mm^3^) for the 500 µm group compared to 0.61/mm^3^ (SD ± 0.02/mm^3^) for the 1000 µm group (*p* = 0.0013).

Regarding the mineral density, the mean mineral density for the scaffold was 818.0 mgHA/cm^3^ (SD ± 52.0 mgHA/cm^3^) for the 500 µm group and 474.0 mgHA/cm^3^ (SD ± 11.0 mgHA/cm^3^) for the 1000 µm group (*p* = 0.0057) (Figure 5c). The mineral density of the struts themselves was measured at 1566.0 mgHA/cm^3^ (SD ± 24.0 mgHA/cm^3^) for the 500 µm group and at 1538.0 mgHA/cm^3^ (SD ± 16.0 mgHA/cm^3^) for the 1000 µm group (*p* = 0.177).

#### 3.1.5. Mechanical Test

**Figure 6:**
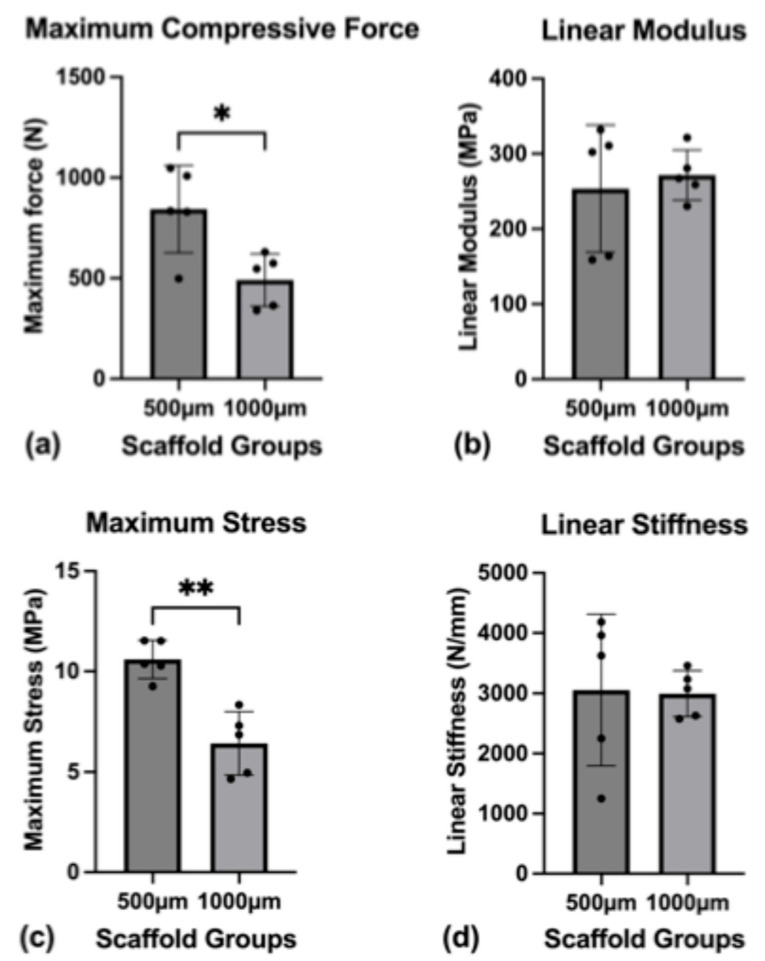
Mechanical characterization. (**a**) Maximum compressive force until scaffold failure; (**b**) linear modulus; (**c**) maximum stress on the scaffolds until failure; (**d**) linear stiffness of the scaffolds. To express a level of significance, the terms * = *p* ˂ 0.05 and ** = *p* ˂ 0.01 were used.

Compression tests were conducted on both scaffold groups to assess the mechanical properties of the scaffolds (Figure 6).

The mean cross-sectional areas of the scaffolds were in a close range of each other, with 76.61 mm^2^ for the 500 µm group and 78.69 mm^2^ for the 1000 µm group (*p* = 0.80). The same was true for the scaffold’s gage (mean gage; 500 µm group: 6.70 mm; 1000 µm group: 6.908 mm) (*p* = 0.3533).

The scaffolds were tested to the point of material failure. The mean maximum compressive force necessary to break the scaffolds was 843.88 N (SD ± 216.62 N) for the 500 µm group and 491.24 N (SD ± 130.24 N) for the 1000 µm group (*p* = 0.0183).

The difference in the mean toe modulus between the groups ((500 µm group: 4.363 MPa (SD ± 1.285 MPa); 1000 µm group: 2.176 MPa (SD ± 0.9701 MPa)) was found statistically significant (*p* = 0.0176), while the mean linear modulus between the groups ((500 µm group: 253.6 MPa (SD ± 84.74 MPa); 1000 µm group: 217.7 MPa (SD ± 33.30 MPa)) was not significantly different (*p* = 0.6739).

The mean maximum stress on the scaffolds was significantly different, with 10.59 MPa (SD ± 0.0564 MPa) for the 500 µm group and 6.424 MPa (SD ± 1.577 MPa) for the 1000 µm group (*p* = 0.0018).

The mean toe stiffness between the two groups ((500 µm group: 52.01 N/mm (SD ± 20.94 N/mm); 1000 µm group: 23.99 N/mm (SD ± 9.664 N/mm)) showed significant differences (*p* = 0.0372) while the mean linear stiffness ((500 µm group: 3053.83 N/mm (SD ± 1256.94 N/mm); 1000 µm group: 2994.44 N/mm (SD ± 380.08 N/mm)) did not differ significantly (*p* = 0.9223).

The mean transition strain for the groups was 8.14% (SD ± 1.45%) for the 500 µm group and 7.82% (SD ± 0.88%) for the 1000 µm group (*p* = 0.6887).

### 3.2. In Vitro Study

#### 3.2.1. Cell Culture

**Figure 7:**
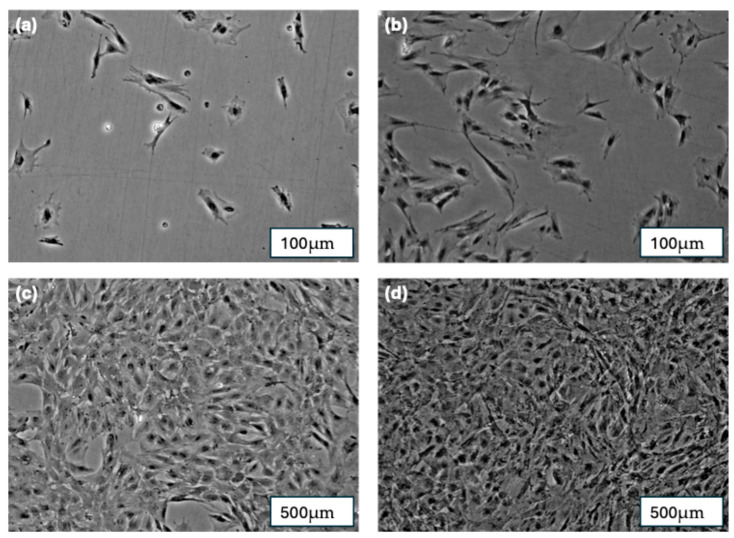
(**a**) pBMSCs after 1 day of cultivation; (**b**) pBMSCs after 5 days of cultivation; (**c**) pBMSCs after 7 days of cultivation; (**d**) pBMSCs after 10 days of cultivation.

On the first day after cell seeding, single spread cells were visible. The cells demonstrated a typical spindle-shaped morphology of pBMSC. After 5 days, cell groups of more than 50 cells (colonies) were formed. The colonies exhibited 80% cell confluency after 7 days of cultivation and achieved nearly 100% confluency after 10 days of cultivation. This is illustrated in Figure 7.

#### 3.2.2. Osteogenic Differentiation Assay of pBMSCs

**Figure 8:**
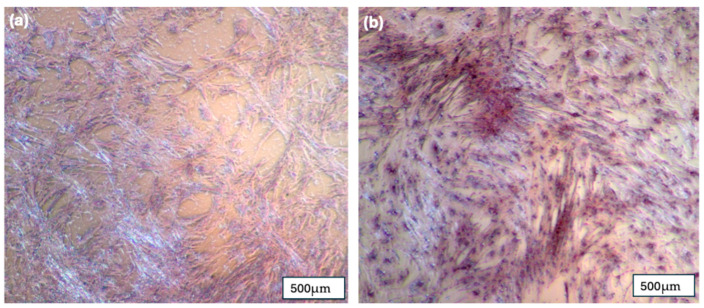
(**a**) pBMSC after 14 days in regular media and stained with 2% Alizarin Red; (**b**) pBMSC after 14 days in osteogenic media and stained with 2% Alizarin Red.

Alizarin Red staining (2%) demonstrated the presence of mineralized calcium deposits (stained red) in the pBMSC cultures after 14 days of exposure to osteogenic media (Figure 8). The formation of these calcium deposits strongly indicates the osteogenic differentiation of the pBMSCs. In contrast, the control group cultured in standard media showed no detectable calcium deposition within the same timeframe.

### 3.3. Cell Count and Viability on the Scaffold Surface

#### 3.3.1. DAPI Staining

**Figure 9:**
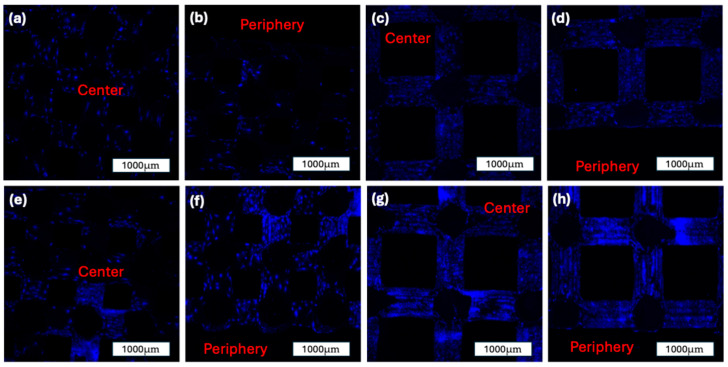
DAPI staining of the two construct groups over time. All images are 2D overlays of n = 120 slices of z-stacks taken via confocal microscopy through a DAPI-Channel: (**a**): 500 µm construct group after 7 days in the ROBS, center of the construct; (**b**): 500 µm construct group after 7 days in the ROBS, periphery of the construct; (**c**): 1000 µm construct group after 7 days in the ROBS, center of the construct; (**d**): 1000 µm construct group after 7 days in the ROBS, periphery of the construct; (**e**): 500 µm construct group after 14 days in the ROBS, center of the construct; (**f**): 500 µm construct group after 14 days in the ROBS, periphery of the construct; (**g**): 1000 µm construct group after 14 days in the ROBS, center of the construct; (**h**): 1000 µm construct group after 14 days in the ROBS, periphery of the construct.

Quantitative DAPI staining was used to assess cell density and spatial distribution across the scaffold surface at two time points (7 and 14 days). Cell nuclei were counted in standardized central and peripheral regions of interest of each construct using Z-stack imaging and 3D image analysis. Representative confocal microscopy images are shown in Figure 9, displaying DAPI-stained nuclei of both scaffold groups over time. All images are 2D overlays of n = 120 slices from Z-stacks acquired through the DAPI channel.

At Day 7, the 500 µm group showed a mean cell count of 4542.33 ± 1623.62 in the central region and 3270.00 ± 968.40 in the periphery. In comparison, the 1000 µm group demonstrated a more uniform distribution, with 4125.50 ± 1089.65 cells centrally and 4221.67 ± 949.61 peripherally. No statistically significant differences were observed between the central and peripheral regions within either group at this time point (*p* = 0.3218 and *p* = 0.9193, respectively).

**Figure 10:**
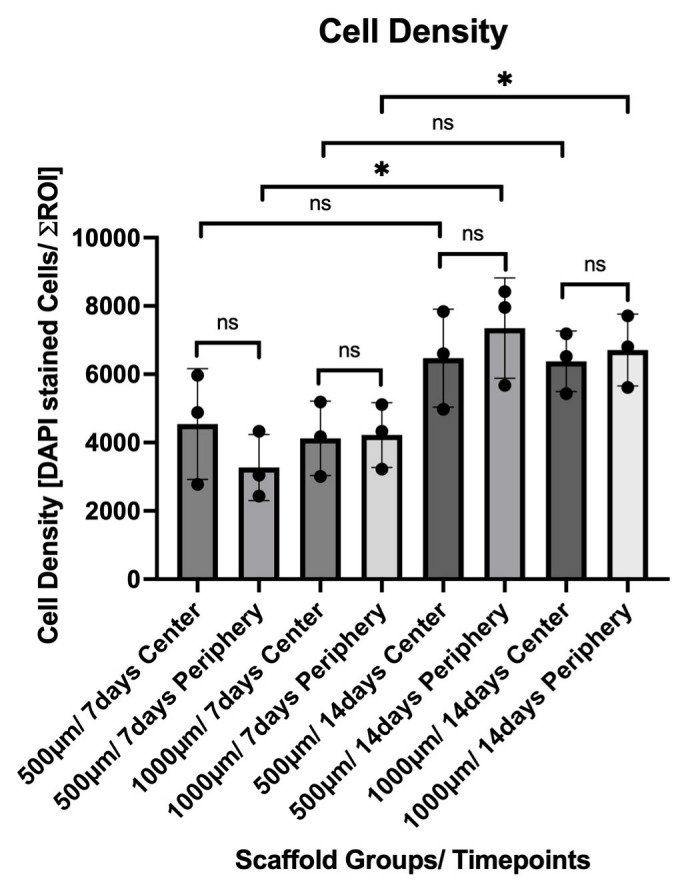
Quantification of DAPI-stained nuclei within the 500 µm and 1000 µm scaffold groups after 7 and 14 days in dynamic culture. Cell counts were measured in standardized central and peripheral regions of interest (ROIs) of each construct (mean ± SD). A significant increase in peripheral cell density was observed from Day 7 to Day 14 within both scaffold groups. Statistical significance: *p* < 0.05 (*); ns = not significant.

By Day 14, both groups exhibited a marked increase in overall cell density. The 500 µm group increased to 6471.08 ± 1435.22 centrally and 7350.50 ± 1468.75 peripherally. A statistically significant increase was noted in the peripheral region compared to Day 7 (*p* = 0.0211). Similarly, the 1000 µm group showed 6380.67 ± 884.50 in the center and 6708.92 ± 1052.15 in the periphery at Day 14, with a significant increase in the peripheral region relative to Day 7 (*p* = 0.0389). The cell density and distribution between the groups are illustrated in Figure 10.

#### 3.3.2. Live/Dead Staining

**Figure 11:**
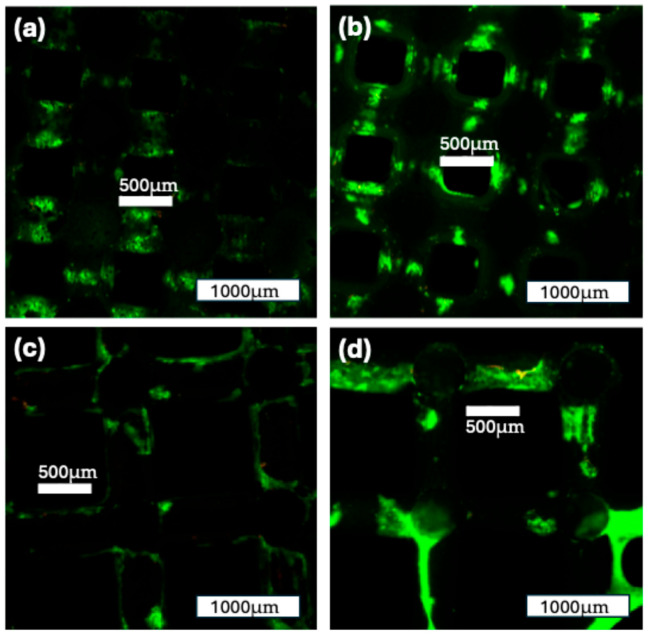
Live/Dead staining of the two construct groups over time: (**a**): 500 µm construct group after 7 days in the ROBS; (**b**): 500 µm construct group after 14 days in the ROBS; (**c**): 1000 µm construct group after 7 days in the ROBS; (**d**): 1000 µm construct group after 14 days in the ROBS; some cell formations can be seen spanning over the macroscopic pores. Green: viable cells; red: nonviable cells.

The staining of the construct with Live/Dead staining showed the formation of cell colonies on the scaffold surface. In both groups, there was an increase in the cell-covered surface area from 7 to 14 days of incubation. The distribution of cell colonies was more heterogeneous after 7 days, and they grew more confluent after 14 days in the ROBS. For both groups and both time points, the number of cells marked with red fluorescence (EthD-1) was low, indicating a low number of dead cells on the surface. Overall, these findings show a high cell viability for both construct groups (Figure 11).

#### 3.3.3. Alkaline Phosphatase (ALP) Activity

**Figure 12:**
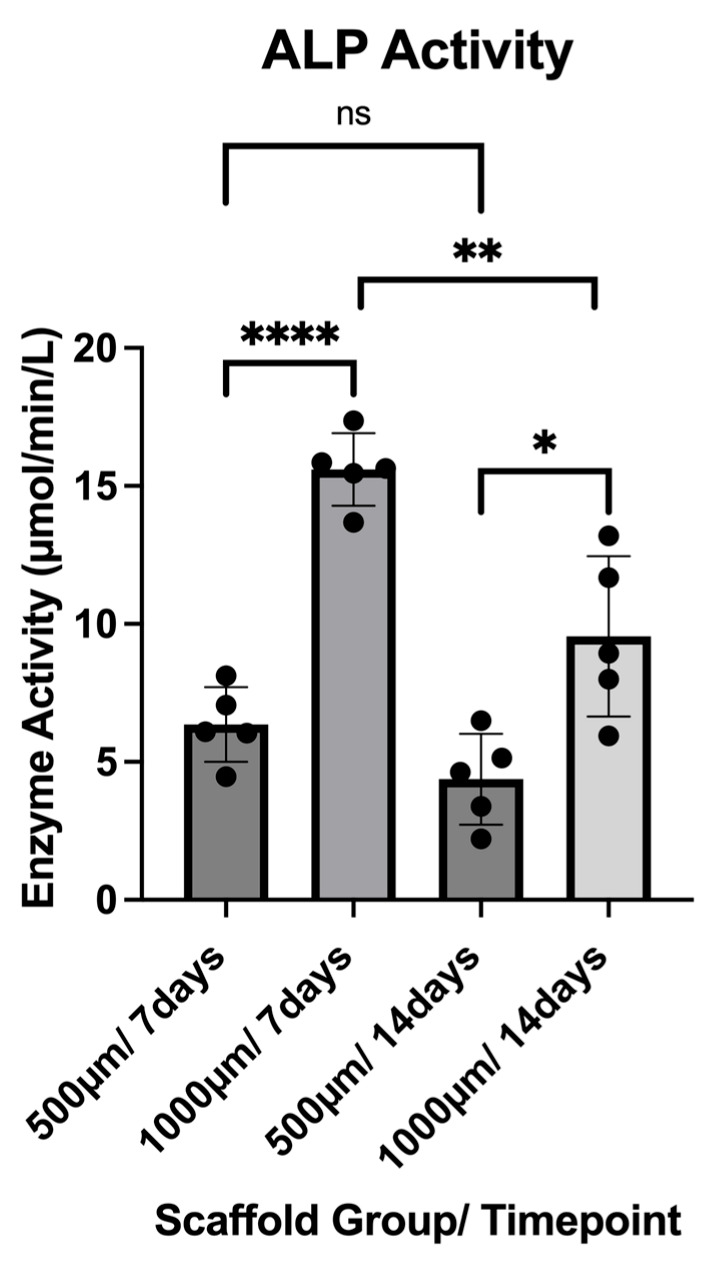
ALP activity of both construct groups (500 µm and 1000 µm) at the timepoints of 7 and 14 days. To express a level of significance, the terms * = *p* ˂ 0.05; ** = *p* ˂ 0.01; **** = *p* ˂ 0.0001 and ns = not significant, were used.

ALP activity was assessed in the culture medium collected from constructs after 7 and 14 days of osteogenic differentiation. Five constructs per group (n = 5) were analyzed, and each sample was measured in technical quadruplicates. The results are reported as µmol/min/L and reflect the enzyme activity present in the supernatant at the time of collection (Figure 12).

At Day 7, the 500 µm group exhibited a mean ALP activity of 6.356 ± 1.356 µmol/min/L, with values ranging from 4.466 to 8.123 µmol/min/L. In contrast, the 1000 µm group showed significantly higher activity at this time point, with a mean of 15.60 ± 1.311 µmol/min/L (range: 13.68–17.37 µmol/min/L; *p* < 0.0001).

At Day 14, a reduction in ALP activity was observed in both groups. The 500 µm group showed a mean activity of 4.372 ± 1.643 µmol/min/L (range: 2.216–6.497 µmol/min/L), while the 1000 µm group maintained higher values with a mean of 9.549 ± 2.900 µmol/min/L (range: 5.937–13.19 µmol/min/L; *p* = 0.0122).

Overall, ALP activity peaked at Day 7 and declined by Day 14 in both scaffold groups, with the 1000 µm group demonstrating significantly greater enzyme activity at both timepoints (500 µm *p* = 0.0721; 1000 µm *p* = 0.0064).

#### 3.3.4. Real-Time Polymerase Chain Reaction (RT-PCR)

**Figure 13:**
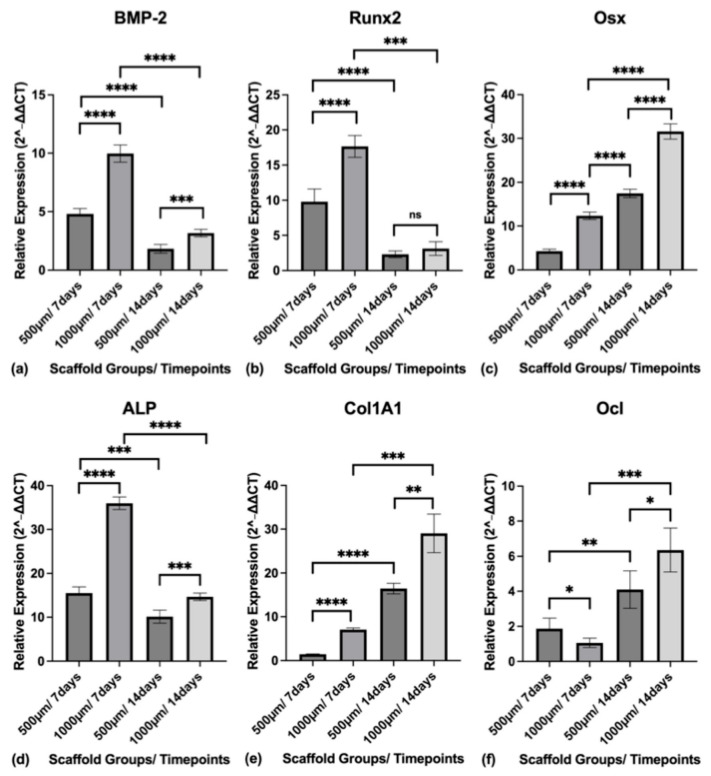
(**a**) Relative expression level of BMP-2 in both construct groups (500 µm and 1000 µm) at the timepoints of 7 and 14 days; (**b**) Relative expression level of Runx2 in both construct groups (500 µm and 1000 µm) at the timepoints of 7 and 14 days; (**c**) Relative expression level of Osx in both construct groups (500 µm and 1000 µm) at the timepoints of 7 and 14 days; (**d**) Relative expression level of ALP in both construct groups (500 µm and 1000 µm) at the timepoints of 7 and 14 days; (**e**) Relative expression level of Col1A1 in both construct groups (500 µm and 1000 µm) at the timepoints of 7 and 14 days; (**f**) Relative expression level of Ocl in both construct groups (500 µm and 1000 µm) at the timepoints of 7 and 14 days. Gene expression levels (2^−ΔΔCT^) for BMP-2, Runx2, Osx, ALP, Col1A1, and Ocl were normalized to GAPDH. To express a level of significance, the terms * = *p* ˂ 0.05; ** = *p* ˂ 0.01; *** = *p* ˂ 0.001; **** = *p* ˂ 0.0001 were used. ns = not significant.

The relative mRNA expression (2^−ΔΔCT^) of osteogenic markers was quantified by qRT-PCR at 7 and 14 days of osteogenic differentiation. Gene expression levels for BMP-2, Runx2, Osx, ALP, Col1A1, and Ocl were normalized to GAPDH and analyzed in both scaffold groups (500 µm and 1000 µm) (Figure 13).

BMP-2 expression was highest at Day 7 in both groups and declined by Day 14. At Day 7, the mean expression was 4.804 ± 0.4560 in the 500 µm group and 9.969 ± 0.7352 in the 1000 µm group (*p* < 0.0001). At Day 14, expression decreased to 1.825 ± 0.3712 and 3.160 ± 0.3168 for the 500 µm and 1000 µm groups, respectively (*p* = 0.0003).

Runx2 expression followed a similar trend. The 500 µm group exhibited a mean expression of 9.788 ± 1.814 at Day 7 and 2.309 ± 0.4736 at Day 14. The 1000 µm group showed significantly higher expression at Day 7 (17.65 ± 1.548; *p* < 0.0001), with a reduction by Day 14 to 3.127 ± 0.9709 (*p* = 0.1428).

Osx expression increased from Day 7 to Day 14 in both groups. In the 500 µm group, expression rose from 4.281 ± 0.4379 to 17.46 ± 0.9704. In the 1000 µm group, the values were higher at both time points, rising from 12.34 ± 0.8548 at Day 7 (*p* < 0.0001) to 31.57 ± 1.751 at Day 14 (*p* < 0.0001).

ALP gene expression peaked at Day 7 and declined by Day 14. The 500 µm group showed 15.49 ± 1.439 at Day 7 and 10.14 ± 1.478 at Day 14. The 1000 µm group demonstrated significantly higher expression with 35.96 ± 1.437 at Day 7 (*p* < 0.0001) and 14.63 ± 0.8460 at Day 14 (*p* < 0.0008).

Col1A1 expression increased significantly from Day 7 to Day 14 in both groups. The 500 µm group increased from 1.463 ± 0.06944 to 16.43 ± 1.223, while the 1000 µm group rose from 7.067 ± 0.3740 (*p* < 0.0001) to 29.04 ± 4.392 (*p* = 0.0021).

Ocl expression also increased with time. At Day 7, the 500 µm group had a mean expression of 1.868 ± 0.5993, while the 1000 µm group showed a lower value of 1.063 ± 0.2652 (*p* = 0.0366). By Day 14, Ocl expression had risen in both groups, reaching 4.100 ± 1.070 in the 500 µm group and 6.354 ± 1.250 in the 1000 µm group (*p* = 0.0160).

## 4. Discussion

### 4.1. Overview and Principal Findings

This study aimed to determine how scaffold pore size influences early osteogenic differentiation of pBMSCs under dynamic in vitro culture conditions. Using a controlled 3D-printed β-TCP scaffold platform, we compared constructs with pore sizes of 500 µm and 1000 µm while maintaining identical material composition and overall design.

Our principal finding is that scaffolds with 1000 µm pores significantly enhanced osteogenic gene expression relative to the 500 µm group, particularly at the early time point of 7 days. Gene expression levels of critical markers such as Runx2, BMP-2, and ALP were markedly upregulated in the 1000 µm group, indicating accelerated osteogenic commitment. Moreover, transcriptional data were supported by functional assays of ALP activity, which also peaked at Day 7 and were significantly higher in the 1000 µm group at both time points. These findings suggest that larger pore sizes, under dynamic perfusion, promote a microenvironment that favors osteogenic differentiation.

Together, these results support the hypothesis that scaffold microarchitecture, and in particular macropore size, plays a critical role in modulating cellular behavior in dynamic culture systems. The superior osteogenic response in the 1000 µm group highlights the importance of pore-scale optimization in designing scaffolds for craniofacial bone tissue engineering.

### 4.2. Interpretation of PCR Results

The quantitative PCR analysis revealed distinct temporal and group-specific patterns in osteogenic gene expression, reflecting the sequential phases of mesenchymal stem cell differentiation toward mature osteoblasts [36,37,38,39,40,41,42]. Early-stage markers such as BMP-2 and Runx2 peaked at Day 7 in both groups, consistent with their established roles in initiating osteogenic differentiation [36,37,38,41]. Notably, expression levels of both genes were significantly higher in the 1000 µm group at this early time point, suggesting a more robust induction of the osteogenic program [36,37,38,41].

Runx2, a master transcription factor critical for early osteoblast lineage commitment, and BMP-2, a potent osteoinductive cytokine, act synergistically during the commitment phase [37,41]. Their enhanced expression in the 1000 µm scaffolds likely reflects improved nutrient and oxygen delivery afforded by the larger pores, which may reduce hypoxic stress and support more efficient gene activation [36,37,38]. This early molecular advantage was further supported by elevated ALP gene expression and enzymatic activity in the same group, both classical indicators of matrix maturation and active osteoblast function [37,42,43].

This early upregulation of osteogenic markers, particularly Runx2, BMP-2, and ALP, is clinically significant, as it suggests the potential to shorten in vitro culture times, accelerating the development of implantable constructs for time-sensitive craniofacial applications such as oncologic and non-oncologic reconstruction. Elevated ALP induction during early in vitro osteogenic culture has been shown to strongly predict in vivo bone-forming potential of human BMSCs, highlighting its value as a surrogate marker for translational graft performance [35].

By Day 14, gene expression of Osx and Col1A1, which are typically associated with intermediate and matrix deposition phases of osteogenesis [38], showed significant increases in both groups, but remained substantially higher in the 1000 µm group.

Ocl, a late-stage marker of mineralizing osteoblasts [39], was lower in the 1000 µm group at Day 7 but showed a more pronounced upregulation by Day 14, exceeding the 500 µm group. This transient pattern is consistent with Ocl’s late position in the osteogenic cascade and with the sequence observed here [39,44,45]. The timing might suggest that, under dynamic perfusion, larger-pore constructs first prioritize early commitment and premineralization activity (Runx2/BMP-2 and ALP), with Ocl upregulation emerging as matrix maturation progresses [46,47,48]. The Day 14 increase in Ocl in the 1000 µm group coincides with higher Osx and Col1A1, which is consistent with the transition toward matrix organization and subsequent mineral deposition [45]. It is also important to note that Ocl mRNA does not necessarily track immediate mineral accrual [49,50]. Protein accumulation and incorporation into the extracellular matrix can lag transcription, particularly in perfused systems where mechanobiological cues might modulate gene timing [49,50]. Accordingly, the lower Ocl at Day 7 in the 1000 µm group likely reflects stage-specific kinetics rather than an attenuated osteogenic response, which becomes evident by Day 14 when Ocl surpasses the 500 µm group [44]. Future work extending the culture period and adding osteocalcin protein assays (e.g., ELISA or immunostaining) alongside quantitative mineralization readouts would better resolve the timing of transcription-to-function under dynamic flow.

These sequential gene expression dynamics, early induction of Runx2 and BMP-2 followed by sustained expression of Osx, Col1A1, and Ocl, are consistent with a coordinated osteogenic differentiation process [38,43,51]. Notably, the consistently higher levels across most markers in the 1000 µm group indicate not only accelerated but also more complete osteogenic maturation under dynamic perfusion.

Collectively, these molecular findings point toward a favorable interplay between scaffold architecture and dynamic fluid flow. The improved transport conditions within the larger-pore scaffolds likely mitigated diffusional limitations, supporting enhanced metabolic activity and transcriptional responsiveness of pBMSCs throughout the osteogenic cascade.

### 4.3. Mechanical and Structural Considerations

Although the 1000 µm pore scaffolds demonstrated superior osteoinductive properties, they exhibited reduced mechanical performance compared to the 500 µm group. Mechanical testing confirmed significantly lower compressive strength in the larger-pore group, primarily due to its higher porosity and lower connectivity density. This trade-off between mechanical integrity and biological activity is a well-established challenge in scaffold design, particularly for load-bearing craniofacial applications such as mandibular reconstruction [52,53].

However, the use of high-resolution 3D-printing technologies offers a critical advantage in navigating this trade-off [15,16]. Additive manufacturing enables the fabrication of scaffolds with highly precise and reproducible microarchitectures, allowing for the deliberate spatial modulation of structural parameters such as pore size, porosity, and strut thickness [15,16]. This opens the door to patient-specific scaffold design strategies that combine mechanical and biological optimization within a single construct [15,16,54,55,56].

For larger bone defects, particularly in anatomically complex regions like the mandible, and even more so in the midface, where structures such as the zygomatic arch, naso-orbito-ethmoid complex, and frontal bone present greater three-dimensional complexity, it is feasible to engineer scaffolds with regional heterogeneity. Denser, smaller-pore domains can be strategically positioned in load-bearing areas to provide structural support, while more porous zones with larger pores can be integrated in regions experiencing lower mechanical stress to enhance osteogenic ingrowth and nutrient exchange [54,55,56]. These bioinspired design principles mimic natural bone, which exhibits functional gradation from dense cortical bone to more porous cancellous structures [54,55,56].

Thus, although 1000 µm pores alone may not offer optimal mechanical strength, the ability to integrate them into a zonally tailored scaffold architecture via advanced 3D printing workflows provides a powerful strategy to reconcile biological performance with clinical functional demands.

To contextualize the reduced resistance of the 1000 µm scaffolds, our measurements should be benchmarked against the properties of natural bone. The ultimate compressive stresses recorded here (500 µm: 10.59 ± 0.0564 MPa; 1000 µm: 6.424 ± 1.577 MPa) sit within reported trabecular-bone ranges (0.1–30 MPa) [57] but remain far below typical cortical-bone strengths (100–230 MPa) [58,59]. Likewise, our apparent (linear) moduli (500 µm: 253.6 ± 84.74 MPa; 1000 µm: 217.7 ± 33.30 MPa) fall in the trabecular regime [57], whereas cortical bone exhibits moduli in the order of tens of GPa [60]. These comparisons suggest that large-pore regions (1000 µm) alone are unlikely to sustain primary load paths; instead, clinical viability under load requires zonal architectures in which smaller-pore, column-like domains distribute physiological forces, while larger-pore zones support rapid tissue ingrowth and perfusion. In their current monolithic form and geometry, both pore designs should therefore be considered load-sharing rather than load-bearing constructs at implantation, necessitating temporary fixation (e.g., reconstruction plates) while new bone forms and remodels within the scaffold.

Additionally, EDX analysis confirmed that both pore-size groups exhibit identical elemental compositions and surface chemistry. This is important, as it ensures that the observed biological differences can be attributed to variations in pore architecture rather than material composition [61,62]. By ruling out compositional effects, the study isolates scaffold design parameters, specifically macropore size, as the primary driver of the divergent osteogenic responses. This strengthens the translational relevance of the findings, since it highlights architecture, a tunable design variable in additive manufacturing, as the key determinant of scaffold performance under dynamic culture.

### 4.4. Role of Dynamic Culture Conditions

Dynamic culture played a pivotal role in enhancing the osteogenic potential of both scaffold groups. The implementation of the ROBS facilitated continuous perfusion, improving nutrient delivery, oxygen availability, and metabolic waste clearance, parameters that are often suboptimal in traditional static cultures [8,63,64,65]. Unlike static systems, where nutrient gradients and localized hypoxia can lead to uneven cell behavior and necrotic zones, the dynamic environment promoted a more physiologically relevant milieu conducive to osteogenic differentiation [8,63,64,65].

A key outcome of this approach was the remarkably uniform distribution of viable cells across the entire scaffold structure. DAPI-based cell quantification revealed a homogeneous spatial distribution of nuclei in both the central and peripheral scaffold regions at both 7 and 14 days, with no significant differences between groups. Similarly, Live/Dead staining confirmed high cell viability throughout the constructs, with very few nonviable cells detected. These findings suggest that dynamic perfusion effectively mitigated central necrosis and supported sustained cell proliferation and survival even in the inner core of the scaffolds [8,63,65,66].

Such homogeneous cell colonization is rarely observed in static cultures, where limited mass transport can result in nutrient depletion and hypoxia in the center of the scaffold [63,65]. Moreover, dynamic flow introduces low levels of mechanical shear stress, a known stimulant for osteogenic signaling, which may have further supported early lineage commitment, especially in combination with favorable scaffold architecture [64,65].

The synergistic interplay between dynamic flow and the 1000 µm macroporous design likely created an optimal biophysical environment, enabling deep perfusion and uniform cellular stimulation. These effects were reflected in both the gene expression profiles and ALP activity data, with the 1000 µm group consistently outperforming the 500 µm group under identical bioreactor conditions [63,64,67].

Altogether, this study underscores the value of dynamic culture systems in bone tissue engineering. By overcoming the diffusion limitations inherent in static systems and enhancing scaffold–cell interaction, dynamic perfusion provides a critical platform for evaluating and optimizing scaffold designs intended for translational applications.

While several previous large animal studies have reported favorable outcomes with smaller or intermediate pore sizes (typically 200–450 µm) [9], our findings clearly demonstrate that, under dynamic in vitro conditions, larger pore sizes (1000 µm) significantly enhance osteogenic differentiation. This apparent discrepancy can be attributed to fundamental differences in experimental design and biological context. Many of the earlier studies relied on static seeding of scaffolds followed by direct implantation into defect sites, often without the use of preconditioning bioreactor systems [9,10]. As a result, smaller pores may have facilitated better initial cell retention or provided a favorable microenvironment for early matrix deposition in vivo, albeit under conditions of limited control over oxygenation, nutrient availability, and fluid flow [63,65].

In contrast, our study employed a controlled ex vivo environment using the ROBS, which ensures continuous media perfusion, enhanced oxygen delivery, and removal of metabolic waste [8,63,64]. Under these dynamic culture conditions, the advantages of larger pore sizes become more pronounced, promoting deeper media penetration, more homogeneous cell distribution, and superior osteogenic gene activation [63]. Notably, our results reflect a pre-implantation phase that mimics modern tissue-engineering strategies, where scaffolds are biologically primed before in vivo application [10].

Furthermore, while recent advances in animal models increasingly involve in vivo bioreactor concepts, such as muscle pouch implantation, these models tend to focus on the biological conditioning of constructs rather than systematic variation of scaffold architecture [10]. Our study provides new insights by isolating and evaluating the impact of pore size under standardized dynamic conditions, offering valuable guidance for scaffold optimization in future translational approaches that combine bioreactor-based priming with customized, load-adapted scaffold designs.

### 4.5. Additional Findings: Cell Viability and Density

Beyond osteogenic differentiation, we assessed fundamental cellular parameters including viability, spatial distribution, and cell density across both scaffold groups and time points. Live/Dead staining demonstrated consistently high cell viability in all constructs, with no apparent regional variations or central necrosis. This observation is particularly noteworthy, as maintaining cell survival in the inner regions of 3D scaffolds is a well-documented challenge, especially in static cultures where mass transport is limited [64,68,69,70].

Quantitative analysis using DAPI staining confirmed that cells were not only viable but also uniformly distributed throughout both the central and peripheral scaffold regions [68,70,71]. Importantly, total cell numbers increased from Day 7 to Day 14 in both groups, indicating ongoing cell proliferation or sustained viability under dynamic perfusion. However, no significant differences in cell numbers were observed between the 500 µm and 1000 µm groups at either time point.

These results suggest that the observed differences in osteogenic marker expression and ALP activity are not attributable to variations in cell proliferation or seeding efficiency but rather reflect genuine differences in cellular differentiation. In other words, the enhanced osteogenic response in the 1000 µm scaffolds cannot be explained by a higher number of metabolically active cells. Instead, it points to qualitative changes in cellular behavior, likely driven by the improved transport dynamics and mechanobiological cues enabled by the larger pore architecture under dynamic flow [63,67].

This distinction underscores the importance of evaluating both cell quantity and functional status in scaffold-based tissue engineering studies. A scaffold may support high cell viability yet fail to promote differentiation if the microenvironment is suboptimal [63]. In our model, dynamic culture conditions combined with favorable scaffold geometry succeeded in achieving both high viability and strong osteogenic activation, a dual requirement for successful clinical translation [9,10,63].

An important consideration is the use of pBMSCs in this study. Porcine cells are widely employed in preclinical bone tissue engineering due to their availability, comparable physiology, and similar remodeling dynamics relative to humans [72,73,74]. Nonetheless, species-specific differences in osteogenic gene expression have been documented. Time-course studies in pBMSCs versus human MSCs show variations in the magnitude and timing of upregulation for canonical markers such as Runx2 and ALP, indicating potential shifts in differentiation kinetics between species [43,75]. Despite these differences, the sequential cascade from early commitment (Runx2, BMP-2) to matrix maturation (Col1A1, Osx) and late mineralization markers (Ocl) is highly conserved across species [76,77,78]. This supports the translational relevance of our findings. Moreover, the use of pBMSCs represents a direct bridge between in vitro scaffold evaluation and subsequent large animal studies, which are often conducted in porcine models [10,73,79,80,81]. Our results therefore provide an important step toward translating scaffold design concepts from in vitro systems into in vivo preclinical testing.

### 4.6. Limitations

While this study provides important insights into the influence of scaffold pore size under dynamic culture conditions, several limitations should be acknowledged. First, only two discrete pore sizes (500 µm and 1000 µm) were evaluated. Although these sizes were selected specifically to reflect clinically relevant extremes, intermediate or gradient pore architectures, which may offer a more balanced compromise between mechanical stability and osteoinductive potential, were not investigated. A future step for subsequent studies should include broader pore size ranges or multi-scale scaffold designs to refine architectural optimization.

Second, the study was conducted entirely in vitro using an ex vivo bioreactor system, which, although well controlled, lacks the full biological complexity of in vivo environments. Specifically, key regenerative components such as host vasculature, immune cell populations, and systemic signaling factors were absent [67,82,83]. While the ROBS system successfully recapitulates aspects of mechanical and nutritional conditioning, it does not model host integration, angiogenesis, or inflammatory responses, all of which influence scaffold performance in clinical applications [67,82,84,85].

Third, while pBMSCs provide a relevant preclinical cell source, species-specific differences in osteogenic marker expression limit direct extrapolation to human cells. Nevertheless, as porcine models are a standard for subsequent large animal validation, our findings represent an important step toward bridging in vitro data with in vivo preclinical translation.

Fourth, while the mechanical characterization confirmed that both scaffold designs fall within the range of trabecular bone [57,59], their strength and stiffness are far below those of cortical bone [60]. In their current monolithic form, these scaffolds should therefore be regarded as load-sharing rather than load-bearing constructs, necessitating additional fixation during implantation.

Fifth, the evaluation was limited to an early time frame of 14 days, focusing on osteogenic gene expression and ALP activity as early indicators of differentiation. While these are established markers of osteogenesis, long-term outcomes such as matrix mineralization, scaffold degradation, and mechanical integration remain unaddressed. Additionally, gene expression data, while informative, do not always correlate linearly with protein activity or functional bone formation [67].

Finally, although the β-TCP scaffolds used in this study are clinically relevant, the results may not directly translate to scaffolds made of different materials or fabrication techniques [85]. Further validation in vivo, particularly in large animal models with critical-sized craniofacial defects, is necessary to confirm the clinical relevance of these findings.

### 4.7. Future Directions

Building on the promising results of this study, several avenues should be pursued to advance scaffold-based bone regeneration toward clinical translation. First, future work should investigate a broader range of pore sizes, including intermediate and multi-scale architectures that may further optimize the balance between mechanical stability and biological performance. Incorporating gradient or zonal pore designs could enable region-specific functions within a single scaffold, such as load-bearing strength in one area and rapid cellular infiltration in another [86,87].

Second, long-term studies are needed to evaluate matrix mineralization, scaffold degradation, and functional integration. Protein-level analyses and imaging-based assessments (e.g., μCT, immunohistochemistry) would provide additional insight into the quality and extent of bone formation. Importantly, in vivo validation in large animal models is essential to confirm that the osteogenic benefits observed under dynamic in vitro conditions translate to effective regeneration in the complex biological environment of a living organism [29].

A particularly pressing consideration in oral and maxillofacial surgery, especially in resection cases, is the need for the timely availability of implantable grafts. Current gold standard procedures, such as autologous bone grafting with microvascular anastomosis, are typically performed in a single-stage operation immediately following tumor resection [2]. This clinical reality underscores the need to produce functional, patient-specific bone constructs within the short window between diagnosis and surgery. Scaffold designs that support rapid osteogenic differentiation of autologous BMSCs and promote early matrix deposition during ex vivo bioreactor culture could be critical in meeting this translational need.

To that end, integrating scaffold engineering with advanced culture systems, including dynamic bioreactors, osteogenic priming protocols, and eventually immune-compatible environments, is key to shortening culture timeframes while enhancing graft performance. These efforts will bring regenerative strategies closer to the clinical goal of generating ready-to-implant, biologically active bone grafts tailored to the anatomical and temporal constraints of complex maxillofacial reconstructions.

Artificial intelligence (AI) is likely to play a significant role in scaffold design moving forward [88], particularly as our results underscore the sensitivity of osteogenic outcomes to pore architecture. Rather than relying solely on empirical testing, AI-driven approaches can integrate datasets on material properties, cellular responses, and anatomical requirements to predict scaffold architectures that optimize both biological and mechanical outcomes [88,89,90,91]. In this context, AI could be especially useful in systematically exploring pore-size distributions, interconnectivity patterns, and a multitude of zonal designs that would be impractical to test experimentally [88,89,92]. Experiments could focus on designs that have proven valid in in silico studies [88].

Furthermore, AI-based tools such as finite element modeling (FEM) and computational fluid dynamics (CFD) are increasingly applied to simulate load distribution and perfusion flow within scaffolds [88,92,93]. These models complement experimental work by allowing in silico evaluation of how structural variations influence nutrient transport and mechanical performance, thereby accelerating scaffold optimization [88,92,94].

From a translational perspective, the combination of AI-guided design with 3D printing and dynamic bioreactor culture could help generate patient-specific constructs within clinically relevant timeframes. This aligns with the needs of craniomaxillofacial surgery, where defect-adapted scaffolds must often be fabricated and preconditioned rapidly between diagnosis and reconstruction [2].

## 5. Conclusions

This study demonstrates that scaffold pore size is a critical determinant of early osteogenic differentiation under dynamic in vitro culture conditions. Specifically, β-TCP scaffolds with 1000 µm pores significantly enhanced the expression of key osteogenic genes (Runx2, BMP-2, ALP, Osx, Col1A1, and Ocl) and ALP enzyme activity compared to scaffolds with 500 µm macropores. These results indicate that larger pores, when combined with dynamic perfusion, create a more favorable microenvironment for osteogenic commitment and maturation.

While the larger-pore scaffolds exhibited reduced mechanical strength, the findings underscore the importance of balancing biological performance with mechanical stability in scaffold design.

Altogether, this work highlights the role of scaffold microarchitecture in directing stem cell fate under dynamic conditions, providing a foundation for optimizing 3D-printed constructs in bone tissue engineering.

## Figures and Tables

**Table 1:** Primer for the RT-PCR.

Gene	Forward (5′-3′)	Reverse (3′-5′)	Reference
ALP	TCAGCTCCACCACAAACCC	GCGTTGGTGTTGTATGTCTTGG	AH012163.1
Runx2	CATCCATCCACTCCACCACC	ACTGAGAGTGGAAGGCCAGA	XM_005666074.3
Osx	CTCATTCCCTGGGCTCAC	TGGGCAGACAGTCAGAAGAG	AY514037
Col1A1	CCCTGCCAGATCTGTGTCTG	GTGGTTTCCTGGTCGGTGG	XM_005668927.1
Ocl	TCAACCCCGACT GCGACGAG	TTGGAGCAGCTG GGATGATGG	AW346755
BMP-2	TGCTGGACCTGTACCGCCGACATTC	GCTGGCATTCGGAGTCACCAACCTG	NM_001195399.1
GAPDH	GGTCGGAGTGAACGGATTTG	AGTGGAGGTCAATGAAGGGG	NM_001206359.1

Abbreviations: Alkaline phosphatase (ALP); Runt-related transcription factor 2 (Runx2); Osteoblast-specific transcription factor/Osterix (Osx); Collagen, type I, alpha 1 (Col1A1); Osteocalcin (Ocl); Bone morphogenetic protein 2 (BMP-2); Glyceraldehyde 3-phosphate dehydrogenase (GAPDH).

## Data Availability

The data presented in this study are available on reasonable request from the corresponding author.
